# IL-4 and IL-17 Are Required for House Dust Mite-Driven Airway Hyperresponsiveness in Autoimmune Diabetes-Prone Non-Obese Diabetic Mice

**DOI:** 10.3389/fimmu.2020.595003

**Published:** 2021-02-11

**Authors:** Anne-Perrine Foray, Céline Dietrich, Coralie Pecquet, François Machavoine, Lucienne Chatenoud, Maria Leite-de-Moraes

**Affiliations:** ^1^ Université de Paris, Paris, France; ^2^ Laboratory of Immunoregulation and Immunopathology, INEM (Institut Necker-Enfants Malades), CNRS UMR8253 and Inserm UMR1151, Paris, France

**Keywords:** asthma, NOD mice, IL-17, iNKT cells, IL-4, house dust mite, iNKT cells, airway hyperreactivity

## Abstract

Allergic asthma is characterized by airway inflammation with a Th2-type cytokine profile, hyper-IgE production, mucus hypersecretion, and airway hyperreactivity (AHR). It is increasingly recognized that asthma is a heterogeneous disease implicating complex immune mechanisms resulting in distinct endotypes observed in patients. In this study, we showed that non-obese diabetic (NOD) mice, which spontaneously develop autoimmune diabetes, undergo more severe allergic asthma airway inflammation and AHR than pro-Th2 BALB/c mice upon house dust mite (HDM) sensitization and challenge. The use of IL-4-deficient NOD mice and the *in vivo* neutralization of IL-17 demonstrated that both IL-4 and IL-17 are responsible by the exacerbated airway inflammation and AHR observed in NOD mice. Overall, our findings indicate that autoimmune diabetes-prone NOD mice might become useful as a new HDM-induced asthma model to elucidate allergic dysimmune mechanisms involving Th2 and Th17 responses that could better mimic some asthmatic endoytpes.

## Introduction

Asthma is an heterogenous immune pathology characterized by wheeze, cough, shortness of breath, chest tightness, and variable degrees of airflow limitation. These symptoms are associated with different patterns of inflammation (1–5). Commonly, asthma is distinguished in two types: allergic and non-allergic. In the first case, inflammation is primarily caused by type 2 immune responses mediated through the Th2 cytokines IL-4, IL-5, and IL-13 and associated with increased Th2 cells and eosinophils in the airways (6, 7). By contrast, non-allergic asthma is mainly triggered by an inflammatory response to viral infections with a major neutrophilic component (7). There is mounting evidence that neutrophilic forms of murine and human asthma are associated with IL-17A (hereafter referred to as IL-17) (8–10).

Th2 and Th17 inflammatory pathways in asthma are currently in investigation (11–13). It was reported that therapeutic targeting of Th2 and Th17 cytokines resulted in the amplification of activity of the opposing pathway (14). The cross-talk between these pathways is complex and further analysis are required to better understand this complexity.

We have previously reported using a classical asthma protocol induced by sensitization and challenge with ovalbumin (OVA) that non-obese diabetic (NOD) mice, which spontaneously develops insulin-dependent diabetes (15, 16), presented an exacerbated Th2-mediated airway inflammation and AHR (17). Further, we reported that NOD mice were prone to produce pro-Th17 cytokines and that IL-17-producing iNKT (iNKT17) cells (18, 19) were overrepresented in these mice (20). These observations led us to examine in more detail whether both Th2 and Th17 inflammatory pathways were implicated in the exacerbated airway inflammation in NOD mice. Here we used a more relevant allergic asthma allergen, the house dust mite (HDM) extracts, and analyzed the airway inflammation by comparing NOD and the Th2 prone BALB/c mice. We show that both IL-4 and IL-17 are critically implicated in the exacerbated AHR observed in NOD mice.

## Materials and Methods

### Mice

Eight to 10-week-old specific pathogen-free NOD and BALB/c mice were bred in our facility. All animal experiments were carried out according to the guidelines for care and use of animals approved by the French Institutional Committee (*APAFIS#4105-201511171831592*).

### Airway Allergen Sensitization and Challenge Model

Mice were immunized by intra nasal (i.n.) injection of 100 μg of HDM extracts (Greer Laboratories, USA) in 0.2 ml saline solution. Mice were then challenged on days 7, 11, and 17 with i.n. HDM (50 µg/mouse) or saline solution. Twenty-four hours after the last challenge, mice were anesthetized with a mixture of ketamine (150 mg/kg) and xylasine (400 µg/kg) and their tracheas were cannulated (tracheostomy with ligation). A flexiVent apparatus (SCIREQ) was used to measure airway-specific resistance (Rn, tidal volume of 10 ml/kg at a respiratory rate of 150 breaths/min in response to increasing doses of aerosolized acetyl-β-methylcholine chloride (methacholine; Sigma-Aldrich). Assessments were performed at least three times and the maximum R value obtained after each dose of methacholine was used for the measure.

Airway inflammation was assessed on cytospin preparations of cells from bronchoalveolar lavage fluid [BALF, 3 x 0.5 ml washes with phosphate-buffered saline (PBS)] that were stained with May-Grünwald/Giemsa (Merck). For some experiments, BALF cells were also analyzed by flow cytometry.

Serum was collected and total IgE and HDM-specific IgG1 were measured by ELISA.

### Lung Histology

Lungs were fixed with 10% formalin *via* the trachea, removed, and stored in 10% formalin. Lung tissues were embedded into paraffin and 3 µm sections were stained with periodic acid Schiff (PAS) using standard protocols and examined with a light microscope.

### Flow Cytometry

BALF or lung mononuclear cells were stained at 4°C in staining buffer (1X PBS, 2% FCS, 2mM EDTA), in the presence of Fc block (2.4G2; BD Biosciences) and analyzed by flow cytometry.

Cells were incubated with CD1d-PBS57-APC tetramers and/or the specific antibodies listed below. For intracellular staining, cells were further fixed with 4% PFA, washed, and permeabilized with 0.5% saponin (Sigma-Aldrich), and then incubated with the anti-cytokine antibodies. The cells were washed and fluorescence was detected using a LSRFortessa (Becton Dickinson). Data were analyzed using the FlowJo 10.4.1 software (Tree Star). **Figure S1** represents the gate strategy used.

Antibodies from BD Biosciences: anti-CD3-FITC (145-2C11), anti-CD45-APC-Cy7 (30F11). Antibodies from BioLegend: anti-CD4-Brilliant Violet 605 (RM4-5), anti-CD69-FITC (H1.2F3), anti-CD8a-Brilliant Violet 785 (53-6.7), anti-TCR Vγ1/Cr4-PE (2.11) [Tonegawa 1986 nomenclature, (21)]. Antibodies from eBioscience: anti-CD44-eFluor450 (IM7), anti-TCRb-AlexaFluor 700 (H57-598), anti-IL-13-PE-eFluor610 (eBio13A), anti-IL-17A-PerCP-Cy5.5 (eBio17B7), anti-IL-4-PE-Cy7 (BVD6-24G2), anti-TCRδ-eFluor450 and -PE-Cy7 (GL3), and Fixable Viability Dye eFluor 506.

### Leucocytes From Lung Tissue

After measurement of airway resistance and collection of BALF, lungs were perfused with PBS, lung tissues were cut into pieces using a gentleMACS Dissociator (Miltenyi Biotec) and treated with collagenase type 4 (Thermo Fischer Scientific) plus DNAse I (Roche). The lymphocyte-enriched fraction was collected at the 35–70% interface of Percoll gradients (GE Healthcare). Cells were immediately stained or stimulated for 4 h with with 10^−8^ M PMA and 1 μg ml^−1^ ionomycin, in the presence of 10 μg ml^−1^ brefeldin A (all from Sigma-Aldrich).

### Measurement of Total IgE and HDM-Specific IgG1 by ELISA

Total IgE was measured in the serum by using a mouse IgE ELISA set (BD Biosciences) according to supplier’s recommendations. An indirect ELISA method was used to measure HDM-specific IgG1 levels in serum samples as previously described by Trompette et al. (22). Briefly, 96-well microtiter plates were coated overnight with 100 µl of HDM at 10 µg/ml in PBS. The next day, 200 µl of blocking solution (1% BSA in PBS) was added to the plate for 2 h at room temperature. Subsequently, 100 µl of serum sample diluted 1:100 and 1:500 in blocking buffer was added to the plate at 4°C overnight followed by goat-anti-mouse IgG1 (Southern Biotech) for 1 h. Then HRP-donkey anti-goat IgG (Santa Cruz) was added to the plate for 1 h at room temperature, followed by the substract TMB. Absorbance was measured at 450 nm using a microplate reader (VersaMax microplate reader, Molecular Devices). No HDM-specific IgG1 was detected in control mice.

### mRNA Expression

RNAs were extracted using the RNeasy Plus Mini Kit (Qiagen) including a DNase treatment. Then RNA was reverse transcribed using the High Capacity RNA-to-cDNA Kit (Thermo Fisher Scientific), according to the manufacturer’s instructions. Primers and probes for real-time PCR were provided by Thermo Fisher Scientific under references: beta-2 microglobulin: Mm00437762_m1; interleukin 5: Mm00439646_m1; interleukin 13: Mm00434204_m1; interleukin 17A: Mm00439618_m1; Mucin 5b (Muc5b): Mm00466391_m1. All reactions were performed in triplicate with TaqMan^®^ Fast Advanced Master Mix according to the supplier’s instructions for a StepOnePlus apparatus (Thermo Fisher Scientific). All data were normalized to the internal standard, namely beta-2 microglobulin expression in each sample, and expressed as relative expression using the ΔΔCt method *versus* the reference sample.

### Statistics

Data are expressed as means ± SEM. The AHR values were analyzed with 2-way repeated measures ANOVA followed by Bonferroni correction as a *post-hoc* test. All other values were analyzed with Mann-Whitney U test. Results were considered significant at a *P* value of 0.05 or less (*p<0.05; **p<0.01; ***p<0.001; ****p<0.0001). Data were analyzed using GraphPad Prism version 6 (GraphPad Software).

## Results

### NOD Mice Display Exacerbated Airway Inflammation in Response to HDM Challenge

**Figure 1:**
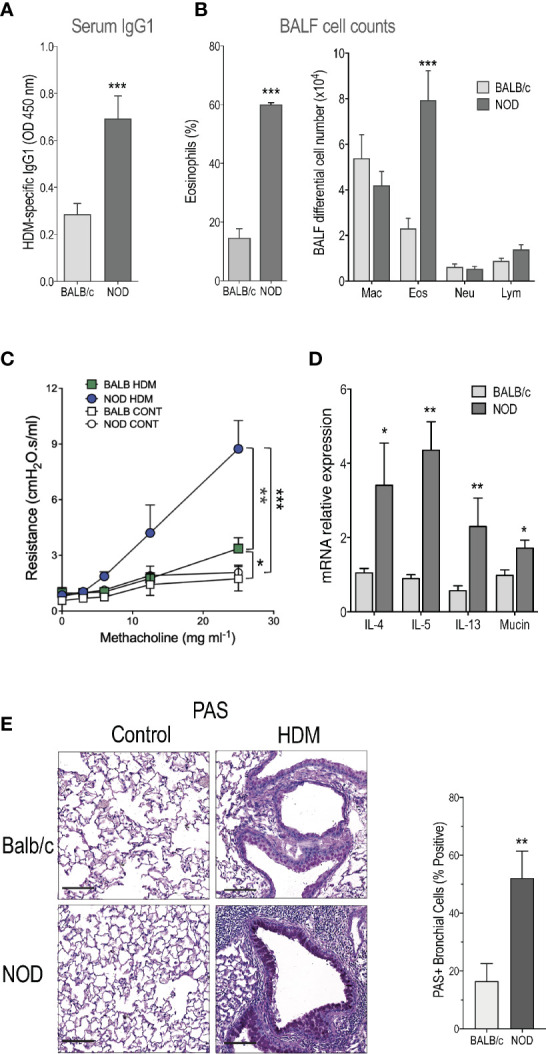
Severe allergic-induced asthma phenotype in non-obese diabetic (NOD) mice. **(A)** Specific IgG1 in house dust mite (HDM)-treated BALB/c and NOD mice (n=9). **(B)** Percentage of eosinophils and macrophage (Mac), eosinophil (Eos), neutrophil (Neu), and lymphocyte (Lym) counts were determined in BALF of HDM-treated BALB/c and NOD mice (n=15). No eosinophils were detected in the lung of control mice. **(C)** Lung resistance was measured 24h after the last challenge with HDM or saline (n=10). **(D)** IL-4, IL-5, IL-13, and mucin messenger RNA (mRNA) expression assessed by quantitative RT-PCR in the lung of HDM-treated BALB/c and NOD mice (n=9). **(E)** Representative PAS-stained lung histology sections of saline controls and HDM-treated BALB/c and NOD mice (N=5). Statistical significance was determined between HDM-treated BALB/c and NOD mice. *p < 0.05, **p < 0.01, ***p < 0.001. Scale bars: 50µm.

Here, we used a HDM-induced asthma model, consisting in intra-nasal (i.n.) immunization followed by three i.n. challenges on days 7, 11, and 17. No adjuvant was added. Mice were sacrificed 24h later. We found that total IgE tended to be higher in NOD compared to the Th2-prone BALB/c mice, but the difference was not statistically significant (1.55 ± 0.13 and 2.12 ± 0.62µg/ml mean ± s.e.m. for BALB/c and NOD, respectively). However, circulating HDM-specific IgG1 was enhanced in NOD compared to BALB/c mice (**Figure 1A**). Another cardinal feature of asthma, the percentage, and the numbers of airway eosinophils were also augmented in NOD mice (**Figure 1B**). No eosinophils, neutrophils, or lymphocytes were observed in BALF from BALB/c or NOD control mice treated with saline solution. Further, we found that NOD mice presented a higher airway hyperreactivity (AHR) as compared to BALB/c mice (**Figure 1C**). Of note, the HDM-induced asthma protocol used here barely induced AHR in BALB/c mice (**Figure 1C**) clearly showing that NOD mice strongly reacted to low doses of HDM sensitization and challenge.

HDM-sensitized and -challenged NOD (hereafter referred to as NOD HDM) mice expressed increased levels of IL-4, IL-5, IL-13, and mucin messenger RNA (mRNA) compared to HDM-sensitized and -challenged BALB/c (BALB/c HDM) mice (**Figure 1D**). In addition, HDM challenge in NOD mice led to increased PAS^+^ goblet cell metaplasia (**Figure 1E**).

### Activation and Cytokine Production by Lung ILT and Conventional T Cells in HDM-Treated BALB/c *Versus* NOD Mice

**Figure 2:**
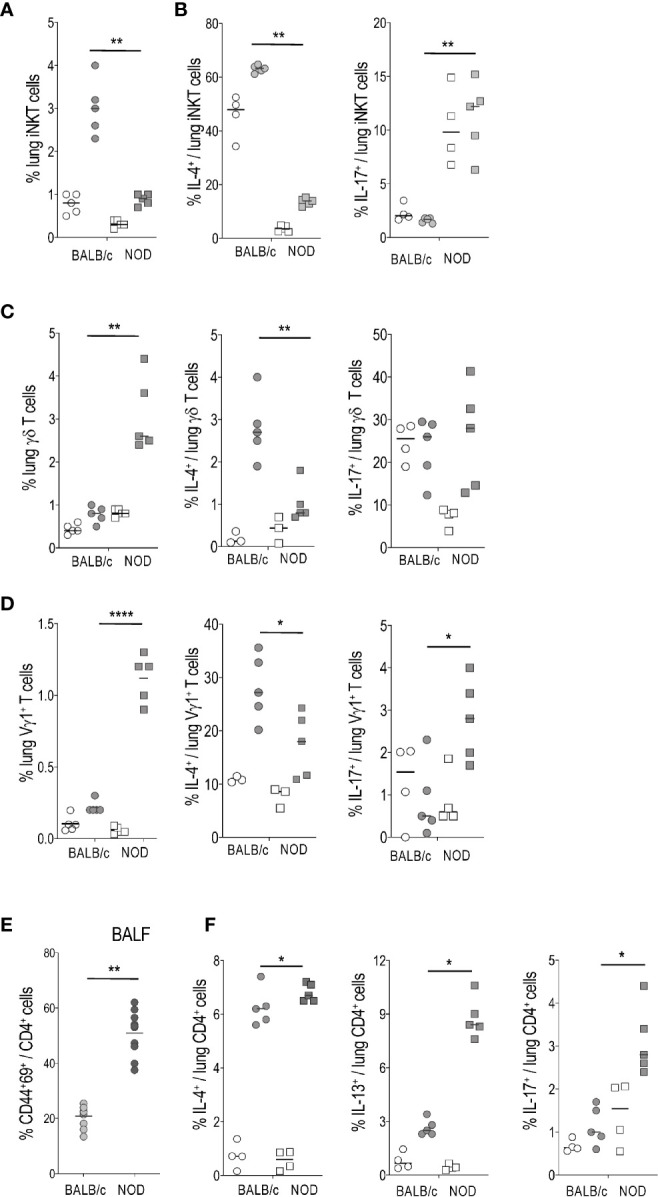
Activation and cytokine production by lung ILT and conventional T cells in house dust mite (HDM)-treated BALB/c *versus* non-obese diabetic (NOD) mice. **(A, B)** Percentage of lung invariant natural killer T (iNKT) cells among gated T cells **(A)** and of IL-4^+^ and IL-17^+^ cells among gated iNKT cells **(B)**. **(C)** Percentage of lung γδ T or Vγ1^+^ γδ T cells among gated T cells. **(D)** Percentage of lung IL-4^+^ and IL-17^+^ cells among gated Vγ1^+^ γδ T cells. **(E)** Percentage of CD44^+^CD69^+^ among gated CD4^+^ T cells in BALF from HDM-treated mice. No CD4+ T cells were observed in BALF from control mice. **(F)** Percentage of lung IL-4^+^, IL-13^+^, and IL-17^+^ cells among gated CD4^+^ T cells. Light circles and squares represent BALB/c and NOD control mice, respectively. Dark circles and squares represent BALB/c HDM and NOD HDM mice, respectively. *p < 0.05, **p < 0.01, ****p < 0.0001.

The distinct cytokine profiles generated during allergic asthma result from activation of both conventional and non-conventional T, also named innate-like T (ILT), cells, such as invariant natural killer T (iNKT) and γδ T. Our previous studies demonstrated that iNKT and γδ T cells contribute to the development of major asthma hallmarks in experimental models (23, 24). Here we provide evidence for reduced iNKT cell frequency in the lung of NOD HDM mice, relative to those recovered from BALB/c mice (**Figure 2A**). The few iNKT cells that did remain in the lung produced less IL-4 than their BALB/c counterpart, but promptly secreted IL-17) (**Figure 2B**), thereby revealing an overrepresentation of IL-17 producers (iNKT17) at the expense of IL-4 producers. These results confirm previous reports showing quantitative iNKT cell deficiency in thymus and spleen of NOD mice (25, 26). Further, our data also confirm that the remaining iNKT cells in NOD mice are mainly iNKT17 (20).

In contrast to iNKT cells, total γδ T cells as well as the Vγ1^+^ γδ T subset, were increased in the lung of NOD HDM mice (**Figures 2C, D**). However, they were similarly biased in favor of a pro-Th17 profile, since total γδ T cells (**Figure 2C**) as well as the Vγ 1^+^ γδ T subset (**Figure 2D**) generated low IL-4 and Vγ1^+^ γδ T subset high IL-17 levels in the lung of NOD HDM compared to BALB/c HDM mice (**Figure 2D**).

Analysis of conventional TCRαβ^+^CD4^+^ T cells revealed that they were more activated in BALF from NOD HDM than from BALB/c HDM mice, as assessed by a higher expression of the CD69 marker (**Figure 2E**). In contrast to non-conventional iNKT and γδ T cells, both Th2 and Th17 profiles were increased among conventional TCRαβ^+^CD4^+^ T cells infiltrating the lung of NOD mice, as demonstrated by a higher proportion of IL-4^+^, IL-13^+^ as well as IL-17^+^ subsets, by comparison with their BALB/c counterpart (**Figure 2F**). These findings indicated an inherent pro-Th2 and pro-Th17 potential in NOD HDM mice.

### Reduced Airway Inflammation in NOD IL-4KO Mice

**Figure 3:**
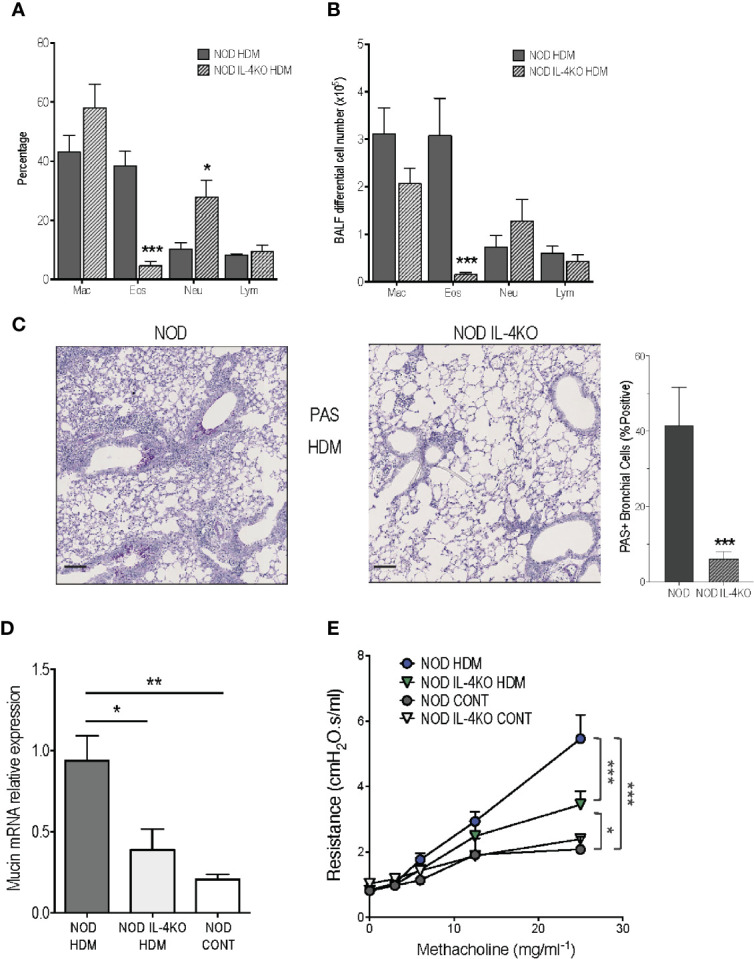
Reduced airway inflammation in non-obese diabetic (NOD) IL-4KO mice. **(A, B)** Percentage and number of macrophages (Mac), eosinophils (Eos), neutrophils (Neu), and lymphocytes (Lym) in BALF of HDM-treated NOD and NOD IL-4KO mice (n=7). **(C)** Representative periodic acid Schiff (PAS)-stained lung histology sections of HDM-treated NOD and NOD IL-4KO mice (N=5). **(D)** Mucin mRNA expression assessed by quantitative RT-PCR in lung of HDM-treated NOD and NOD IL-4KO and NOD control mice (n=9). **(E)** Lung resistance was measured 24h after the last challenge with HDM challenge or saline (n=10). *p < 0.05, **p < 0.01, ***p < 0.001. Scale bars: 50µm.

Knowing that HDM model is globally considered as IL-4 dependent in BALB/c mice (27–29), we first assessed the implication of this cytokine in the severe airway inflammation observed In NOD HDM mice. We addressed the role played by IL-4-producing cells in aggravating asthma, using NOD IL-4KO mice sensitized and challenged with HDM (NOD IL-4KO HDM). In the absence of IL-4, these mice were unable to mount high airway eosinophilia but presented a higher frequency of neutrophils in BALF (**Figures 3A, B**). Further, their expression of mucin mRNA and of PAS^+^ goblet cells were decreased, compared to NOD HDM mice (**Figures 3C, D**). It is noteworthy that AHR was lower in NOD IL-4KO HDM than in NOD HDM mice (**Figure 3E**). However, AHR observed in NOD IL-4KO HDM remained higher than in control saline treated NOD or NOD IL-4KO mice, suggesting the implication of additional mechanisms.

### IL-4 and IL-17 Are Required for the Development of Airway Inflammation in NOD Mice

**Figure 4:**
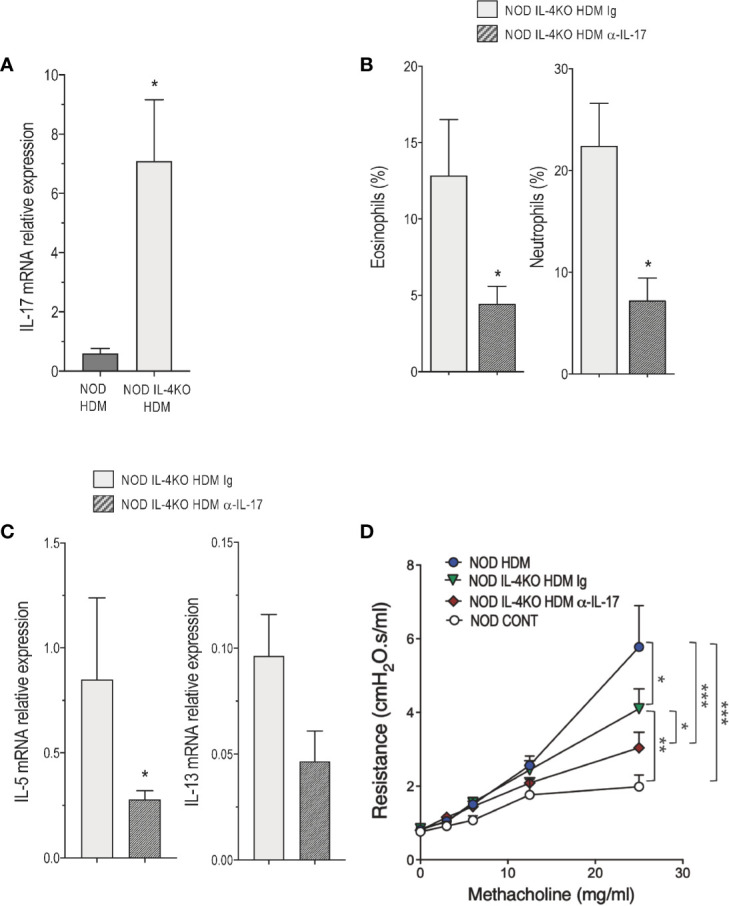
IL-4 and IL-17 are required for the development of airway inflammation in non-obese diabetic (NOD) mice. **(A)** IL-17 messenger RNA (mRNA) expression assessed by quantitative RT-PCR in lung of HDM-treated NOD and NOD IL-4KO mice (n=4 to 6). **(B)** Percentage of eosinophils and neutrophils in bronchoalveolar lavage fluid (BALF) of house dust mite (HDM)-treated NOD IL-4KO mice treated with anti-IL-17 or Ig control (n=6 to 9). **(C)** IL-5 and IL-13 mRNA expression assessed by quantitative RT-PCR in lung of HDM-treated NOD IL-4KO mice treated with anti-IL-17 or Ig control (n=4 to 6). **(D)** Lung resistance was measured 24h after the last HDM challenge or controls (NaCl) (n=6 to 8). *p < 0.05, **p < 0.01, ***p < 0.001.

**Figure 2B** shows that the frequency of iNKT17 cells were higher in the lung of NOD compared with Balb/c mice even in the absence of HDM administration, supporting our previous observation that NOD mice are enriched in these cells (20). Bearing in mind that IL-17 is involved in certain severe cases of asthma likely by regulating neutrophilic inflammation (10) together with our present observation that Vγ1^+^ γδ and CD4^+^ T cells from the lung of NOD HDM mice produced high levels of this cytokine (**Figure 2**), and that NOD IL-4KO HDM mice presented higher levels of neutrophils in the airway (**Figures 3A, B**), we further examined whether IL-17 contributed to the airway inflammation and AHR observed in NOD IL-4KO HDM mice. IL-17 mRNA expression was enhanced in the lung of NOD IL-4KO HDM compared to NOD HDM (**Figure 4A**) supporting the possible implication of IL-17 in the residual inflammation observed in NOD IL-4KO HDM mice. To assess IL-17 implication, NOD IL-4KO HDM mice were treated *in vivo* with a neutralizing anti-IL-17 mAb. These animals developed lower airway eosinophilia and neutrophilia compared to Ig treated NOD IL-4KO HDM mice (**Figure 4B**). Additionally, anti-IL-17 treatment significantly decreased IL-5 and tended to decrease IL-13 mRNA expression in the lung of NOD IL-4KO HDM mice (**Figure 4C**). Finally, AHR was decreased in NOD IL-4KO HDM mice when compared to both NOD IL-4KO HDM Ig and NOD HDM WT (**Figure 4D**). No significant difference was observed between NOD IL-4KO HDM treated with anti-IL-17 and NOD control, indicating that both IL-4 and IL-17 were required for increasing AHR. These results agree with previous observations showing that anti-IL-17 treatment could dampen neutrophil influx in BALF and airway hyperreactivity in mice (30).

## Discussion

Autoimmunity and allergy are two major examples of dysimmune diseases. They are both caused by an uncontrolled immune response against self or non-self-antigens involving Th1 or Th2 mechanisms, respectively. This notion has become more complex since it turned out that Th17 cells could also play a part in this process. NOD mouse spontaneously develops insulin-dependent diabetes, a prototypic Th1-mediated autoimmune disease (31, 32). These animals are also prone to produce pro-Th17 cytokines (20). These observations led us to examine in more detail the inflammatory response of NOD mice in a typical Th2-mediated disease.

Here we demonstrated that NOD mice presented enhanced airway inflammation and AHR in response to HDM sensitization and challenge when compared to the Th2-prone BALB/c mice. Both IL-4 and IL-17 were required for the severity of the symptoms. Our previous report showed that NOD mice developed a more pronounced Th2-mediated inflammatory response to OVA-alum sensitization and challenge compared to BALB/c mice (17). Using an HDM-induced asthma protocol, where HDM extracts were administrated intranasally and without adjuvants in mice, we could get inside the mechanisms implicated in the exacerbated airway inflammation and AHR observed in NOD mice. In fact, we demonstrated, by using NOD IL-4KO mice and by blocking IL-17 *in vivo*, that both Th2- and Th17-mediated immune responses were implicated.

Conventional CD4^+^ T cells were the major source of Th2 cytokines while iNKT, Vγ1^+^, and CD4^+^ produced IL-17 in the lung of NOD mice. We previously reported that iNKT cells were implicated in airway eosinophilia observed in NOD mice sensitized and challenged with OVA but the mechanisms remained to be determined (17). Here we showed that the frequency and the IL-4-producing capacity of iNKT cells in the lung of NOD mice were impaired. The ability of remaining iNKT cells to produce high levels of IL-17 suggest that iNKT17 cells could contribute to airway inflammation, as described (18), and AHR observed in NOD mice. It is noteworthy that both iNKT and Vγ1^+^ T cells, in contrast to conventional CD4^+^ T cells, do not recognize peptides (33–35). These ILT cells can promptly produce cytokines following TCR-dependent or -independent activation (33–35). Taken these findings into account, we could consider that the exacerbated airway inflammation and AHR observed could result from a simultaneous and combined activation of conventional and ILT cells in the lung of NOD mice.

Previous studies reported that Th2 and Th17 inflammatory pathways are reciprocally regulated in asthma (14). Here we confirm these results since NOD IL-4KO HDM presented higher IL-17 mRNA expression and airway neutrophilia than NOD HDM animals indicating that in the absence of IL-4, IL-17 could induce a compensatory airway inflammation in NOD mice. Further, IL-17 blockage sufficed to reduce airway neutrophilia, IL-5 and IL-13 lung mRNA expression and AHR in NOD IL-4KO HDM mice. It was already described that IL-17 blockage could impair both neutrophilia and airway smooth muscle contraction in response to HDM sensitization and challenge in BALB/c mice (30). Our findings clearly indicate that Th2 and Th17 inflammatory responses contribute to asthmatic airway inflammation and AHR observed in NOD mice. However, further studies are required to better determine whether Th2 and Th17 responses act together or independently to induce huge airway inflammation and AHR in NOD mice.

Studies in patients indicated positive associations between asthma and type 1 diabetes (36, 37). However, the mechanisms implicated are still unclear. The co-occurrence of asthma and type 1 diabetes is in direct opposition to the proposed inhibitory model where Th1 and Th2-mediated immune responses could be exclusive. Our present findings support the idea that pathologies associated with pro-Th1, pro-Th2, and pro-Th17 immune responses could co-exist since NOD mice, which spontaneously develop autoimmune diabetes, presented high Th2 and Th17 airway inflammatory responses. Therefore, NOD mice could represent a unique model to better understand the key mechanisms implicated in the association between allergic airway inflammation and autoimmune diabetes. Future studies are required to determine for instance, as reported in humans (37), whether the asthma protocol using HDM sensitization and challenge could modify the incidence of diabetes in NOD mice.

## Conclusion

In summary, we show that NOD mice, which spontaneously develop autoimmune diabetes, undergo more severe allergic asthma airway inflammation and hyperreactivity than pro-Th2 BALB/c mice upon HDM sensitization and challenge. Our data support the conclusion that increased secretion of both IL-4 and IL-17 by pulmonary conventional CD4+ and innate-like T lymphocytes is the major cause of this exacerbated airway inflammation leading to increased severity in NOD mice. We identified iNKT, Vγ1^+^, and CD4^+^ T cells as sources of IL-17 and Th2 cells as IL-4 producers in the lung of HDM NOD mice. In our NOD HDM model, both eosinophils and neutrophils were recruited into the airways. It has already been reported that asthmatic patients whose airways are infiltrated with both eosinophils and neutrophils suffer from more severe symptoms than those recruiting either eosinophils or neutrophils (3, 4, 7). It is our belief that autoimmune diabetes-prone NOD mice might become useful as a new HDM-induced asthma model to elucidate allergic dysimmune mechanisms involving Th2 and Th17 responses that could better mimic some asthmatic endoytpes observed in patients in association or not with autoimmune diseases.

## Data Availability Statement

The raw data supporting the conclusions of this article will be made available by the authors, without undue reservation.

## Ethics Statement

The animal study was reviewed and approved by French Institutional Committee (APAFIS#4105-201511171831592).

## Author Contributions

ML-d-M and LC designed the research. A-PF, FM, CD, and CP performed the research. A-PF and ML-d-M analyzed the data. A-PF, ML-d-M, and LC wrote the manuscript. All authors contributed to the article and approved the submitted version.

## Conflict of Interest

The authors declare that the research was conducted in the absence of any commercial or financial relationships that could be construed as a potential conflict of interest.

## Supplementary Material

Supplementary Figure S1 Flow cytometric gating strategy used to identify Vg1^+^, iNKT (TCRb^+^ CD1d-PBS57^+^), CD4^+^ (CD4^+^TCRb^+^ iNKT^-^) cells from lung and their ability to produce IL-4, IL-13 or IL-17.

## Abbreviations

AHR, airway hyperreactivity; BALF, bronchoalveolar lavage fluid; HDM, house dust mite; ILT, innate-like T cells; iNKT, invariant natural killer T cells; NOD, non-obese diabetic.
